# A Rare Cutaneous Manifestation: Leukocytoclastic Vasculitis after Pfizer-BioNTech COVID-19 Vaccination

**DOI:** 10.1155/2022/4267930

**Published:** 2022-12-31

**Authors:** Yaritza Serrano Gomez, Brittney Grella, Hongbei Wang

**Affiliations:** ^1^Department of Family Medicine, Northwell Health Glen Cove Hospital, Glen Cove, New York, USA; ^2^Department of Medicine, Northwell Health Huntington Hospital, Huntington, New York, USA; ^3^Department of Pathology, Northwell Health, New Hyde Park, New York, USA

## Abstract

There is growing evidence that vaccines against SARS-CoV-2 can cause various skin reactions, many of which have autoimmune origins. These specific vaccine-induced autoimmune conditions with cutaneous manifestations include lupus erythematosus, bullous pemphigoid, vitiligo, alopecia areata, and leukocytoclastic vasculitis (LCV). In particular, LCV, which is also called hypersensitivity vasculitis, is an inflammation of small blood vessels. We present a case of an 81-year-old male evaluated in the emergency department for a bilateral purpuric non-blanching rash that appeared ten days after receiving the Pfizer-BioNTech booster vaccine against SARS-CoV-2. Results of a skin biopsy indicated LCV, and the rash completely resolved three weeks after clinical presentation.

## 1. Introduction

Vasculitis refers to inflammation of blood vessels leading to tissue destruction with or without organ damage. Vasculitis is classified as small, medium, or large vessel vasculitis and may be either idiopathic or associated with an underlying disease [1]. Leukocytoclastic vasculitis (LCV) is a small-vessel vasculitis characterized histopathologically by immune complex-mediated vasculitis of the dermal capillaries and venules [1, 2]. This condition can be idiopathic or associated with infections, neoplasms, autoimmune disorders, certain drugs, and the administration of vaccines, including those for influenza, hepatitis B virus (HBV), Bacille Calmette–Guérin (BCG), and human papillomavirus (HPV) [3]. LCV has been also identified as a rare extraintestinal skin manifestation of ulcerative colitis and more commonly occurs after the UC diagnosis in 59% of cases, and 68% of patients have active intestinal disease at the time of LCV diagnosis [4]. Clinical features of LCV include palpable purpura, small vessel inflammation, and extracutaneous involvement in approximately 30 percent of affected individuals [3]. In this report, we present a case of LCV after the patient received the Pfizer-BioNTech COVID-19 vaccine.

## 2. Case Presentation

**Figure 1:**
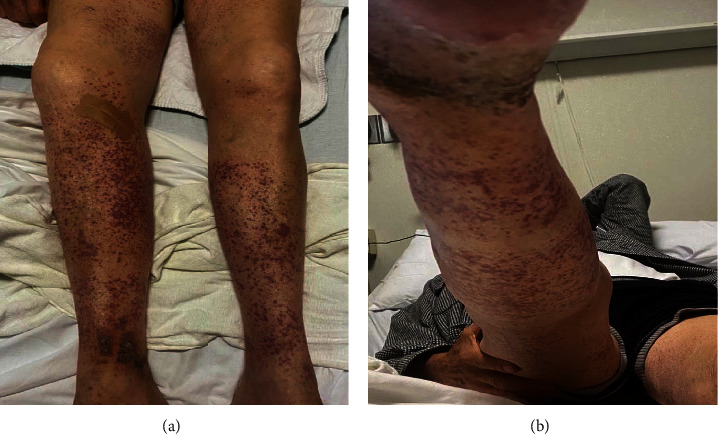
(a, b) Purpuric 1–3 macules located in the lower extremities extending to the anterior and medial thighs.

An 81-year-old male with a past medical history of atrial fibrillation, hypertension, and hypothyroidism presented to the emergency department for evaluation of a bilateral lower extremity purpuric non-blanching rash that appeared two days prior. He had received the Pfizer-BioNTech booster vaccine against SARS-CoV-2 ten days before developing the rash. He reported having a similar rash after the second dose of the Pfizer-BioNTech COVID-19 vaccine six months before evaluation that was self-limited and resolved without further intervention. Physical examination was remarkable for purpuric 1 to 3 mm macules, some barely palpable and located in the shin and anterior and medial thighs of the lower extremities (Figures 1(a) and 1(b)). There was sparing of the abdomen, back, chest, upper extremities, and neck. Initial laboratory tests showed WBC 9.35 × 10^9^/L, Hgb 13.3 g/dL, HCT 41.9%, Na 137 mEq/L, K 3.9 mmol/L, Cr 1.09 mg/dL, AST 18 U/L, ALT 16 U/L, CRP 9 mg/L, ESR 16 mm/hr, C3 120 mg/dL, and C4 20 mg/dL. Urinalysis was normal and without evidence of proteinuria or microhematuria. Additional laboratory tests were negative and included cANCA, pANCA, ANA, Hep C, and B serologies, syphilis screen, and Lyme antibodies. Ultrasound of the lower extremities was also performed and showed no evidence of venous thrombosis. Patient home medications included aspirin, finasteride, levothyroxine, and metoprolol tartrate. He denied the use of any new medications and any additional symptoms, including abdominal pain, vomiting, nausea, melena, hematochezia, hematuria, myalgia, arthralgia, fatigue, headache, fever, and chills.

**Figure 2:**
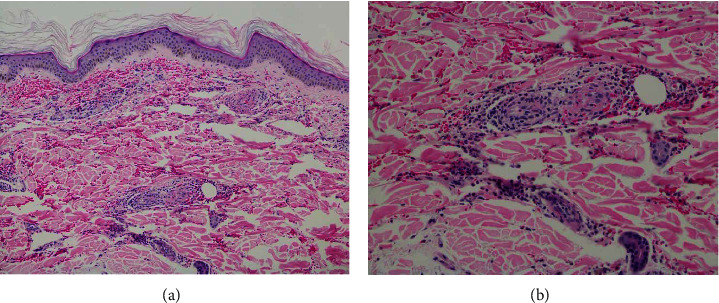
(a, b) 100x low-power image (a) and 200x medium-power image (b) showing extravasation of erythrocytes, perivascular infiltrate of neutrophils, nuclear dusts, and eosinophils, and features of leukocytoclastic vasculitis.

The patient was admitted to the medicine floor for further investigation of the bilateral lower extremities' purpuric rash. During hospitalization, the dermatology and rheumatology specialists evaluated the patient and recommended performing a skin biopsy. A skin biopsy was performed and showed extravasation of erythrocytes, perivascular infiltrate of neutrophils, nuclear dust, and eosinophils, consistent with LCV and negative direct immunofluorescence (Figures 2(a) and 2(b)). The consensus decision was to treat conservatively without the use of systemic corticosteroids or any additional treatment and the patient was discharged home. In the outpatient follow-up, the patient reported complete resolution of the rash three weeks after the symptoms first appeared, and there was no need for any further testing or treatment.

## 3. Discussion

LCV is the histopathologic descriptor of a common form of small-vessel vasculitis involving arterioles, capillaries, and postcapillary venules [5]. The disease can be confined to the skin or affect many organs, including the kidneys, central nervous system, heart, gastrointestinal tract, and lungs [6]. The incidence of cutaneous LCV ranges from 15 to 38 cases per million annually in the United States and appears to affect both sexes and all ages equally, although some studies have noted a slightly higher incidence in males and older individuals [5]. LCV presents as erythematous macules with palpable purpura that involve primarily the lower extremities, but dependent areas, such as the back and buttocks, may also be affected [3, 5]. Patients may be completely asymptomatic or complain of burning, itching, or pain in the involved skin. Systemic symptoms can appear in approximately 30% of patients and may include low-grade fever, malaise, weight loss, myalgias, arthralgias, blood in the urine or stool, abdominal pain, vomiting, cough, numbness, and weakness [7]. The cutaneous manifestations of LCV usually appear approximately 1 to 3 weeks after the triggering event and usually resolve over 2 to 3 weeks [3, 5].

Although history and physical examination are sometimes sufficient to establish a working diagnosis, systematic laboratory tests are usually warranted to perform the differential diagnosis [5]. Tests should include analysis for infectious diseases (e.g., HBV, HCV, and human immunodeficiency virus), serum protein electrophoresis, immunoglobulins (IgG, IgA, and IgM), an antinuclear antibody panel, rheumatoid factor, serum C3, and C4 complement levels, ANCAs, and cryoglobulins [5, 6]. The cornerstone of LCV diagnosis is a skin biopsy with direct immunofluorescence, which should be performed whenever possible for confirmation [3]. After diagnosis, the recommended treatment depends on two major factors: the etiology and the extent of the disease. If LCV is limited to the skin, the management strategy should primarily focus on symptomatic relief since most acute episodes of cutaneous small-vessel vasculitis are self-limited and do not recur, even without treatment [3, 5]. When the cause of LCV is obvious, such as infection or drugs, eliminating or treating the trigger, whenever possible is crucial and often sufficient. In contrast, treatment should generally include systemic corticosteroids when symptoms are severe, intractable, or recurrent [5].

Half of all cases of LCV are idiopathic but secondary causes such as infections and medications have been proposed as the most common triggers [7]. The medications commonly known to cause LCV are beta-lactams, erythromycin, clindamycin, quinolones, vancomycin, sulfonamide, furosemide, allopurinol, NSAIDs, amiodarone, beta-blockers, TNF-alpha inhibitors, SSRIs, metformin, and warfarin [2, 8]. In addition, several vaccines, including those against influenza, HBV, BCG, and HPV, have been implicated in the development of LCV [7, 9]. Including the case detailed here, there have been a few case reports of the development of LCV after administration of the Moderna and Pfizer-BioNTech COVID-19 vaccines [7]. The mechanism by which the SARS-CoV-2 vaccine induces this type of vasculitis is not completely clear, but could potentially be driven by off-target immune activation after vaccination [7, 9]. The SARS-CoV-2 vaccines are DNA/RNA-based vaccines that contain recombinant adenoviral vectors encoding the spike protein of SARS-CoV-2, stabilizers, and immune adjuvants [10]. It is possible that molecular mimicry might develop between the peptides that are expressed in the viral spike protein and host endothelial cells [10, 11]. It has been suggested that the SARS-CoV-2 spike protein can activate the alternative complement pathway, thereby leading to the inflammation of the cutaneous microvasculature.

Recent studies show evidence that the administration of the Moderna and Pfizer-BioNTech COVID-19 vaccines can lead to the development of cutaneous manifestations. These cutaneous manifestations ranged in a wide spectrum, including chilblain‐like, urticarial eruptions, vesicular, morbilliform eruptions, livedoid, delayed large reactions, local injection site reactions, and vasculitis lesions [12, 13]. Delayed large local reactions are mostly found, followed by local injection site reactions, urticarial eruptions, and morbilliform eruptions [12, 13]. Additionally, fewer common reactions include pernio, cosmetic filler reactions, zoster, herpes simplex flares, and pityriasis rosea-like reactions [13]. Recent data shows that approximately 20% of patients receiving the Pfizer-BioNTech COVID- 19 vaccine reported skin reactions after the first dose only, 74% after the second dose only, and 6.0% after both doses, but there is insufficient evidence after the booster dose. The case presented here shows some new evidence of cutaneous manifestation after the booster vaccination. The patient in this case didn't have any systematic symptoms such as fever, abdominal pain, fever, arthralgia, or renal failure at the time of presentation, ruling out other forms of systemic vasculitis including IGA vasculitis, and suggesting more a cutaneous limited form of vasculitis like LCV. Although vasculitis development after SARS-CoV-2 vaccine administration could be worrisome, data suggest that cutaneous reactions to SARS-CoV-2 vaccination are generally minor and self-limited and should not discourage vaccination [13].

## 4. Conclusions

Vaccines against COVID-19 represent a pivotal and effective countermeasure to control the COVID-19 pandemic. This case reveals a potential association between the administration of a COVID-19 vaccine and the development of vasculitis. This could be secondary to complement activation or autoimmunity provoked by the vaccine's SARS-CoV-2 spike protein, leading to inflammation of the cutaneous microvasculature and resulting in vasculitis [3]. Although vasculitis may not be considered a contraindication of vaccination, further investigation is necessary to clarify the characteristics of vasculitis following COVID-19 vaccination.

## Data Availability

All the data generated or analyzed during this study are included in this article. Further enquiries can be directed to the corresponding author.
